# Systemic sclerosis-related digital ulcers; a pilot study of cutaneous oxygenation and perfusion

**DOI:** 10.1093/rheumatology/keaa280

**Published:** 2020-08-23

**Authors:** Elizabeth Marjanovic, Tonia L Moore, Joanne B Manning, Graham Dinsdale, Sarah Wilkinson, Mark R Dickinson, Ariane L Herrick, Andrea K Murray

**Affiliations:** 1 Division of Musculoskeletal and Dermatological, Salford Royal NHS Foundation Trust, Manchester Academic Health Science Centre; 2 Photon Science Institute; 3 Department of Physics and Astronomy, The University of Manchester, Manchester, UK

**Rheumatology key message**

- Non-invasive imaging techniques demonstrate that SSc-related digital ulcers can be hypoxic as well as poorly perfused.

Dear editor, Systemic sclerosis (SSc)-related finger or toe (digital) ulcers (DU) affect up to 50% of patients with SSc [1, 2]. DUs can be chronic, difficult to treat and have a huge impact on quality of life, affecting hand function [3]. Relatively little is known about their pathophysiology. A recent study indicated that both fingertip and extensor surface ulcers are ischaemic [4]. Non-invasive imaging demonstrates increased blood flow with ulcer healing, supportive of reversible ischaemia [4,5]. A small number of case reports documenting SSc-related ulcers responding well to hyperbaric therapy suggest that hypoxia contributes to pathophysiology [6, 7]. It has recently become possible to measure skin oxygenation non-invasively, affording the potential to measure changes in the degree of hypoxia with healing and in response to treatment [8]. The objectives of this small pilot study were to use non-invasive imaging techniques to gain further insight into the pathophysiology of SSc-related DU in terms of cutaneous blood oxygenation levels. The hypotheses of the study were that digital ulcers are hypoxic as well as poorly perfused and that relationships exist between the levels of perfusion and oxygenation and the size of the lesion.

Nine patients with SSc-related DU [one male, eight female, median age 62 (interquartile range 60–69) years, median disease duration since onset of first non-Raynaud’s feature 17 (12–22) years, median duration of Raynaud’s 18 (12–27) years, six limited cutaneous SSc, two anti-Scl70 positive, five anticentromere antibody positive] underwent imaging of one finger ulcer. Images were taken to include the site of ulceration, adjacent skin) and a site away from the ulcer (Fig. 1).

**Fig. 1:** Images showing examples of finger ulcers and associated data 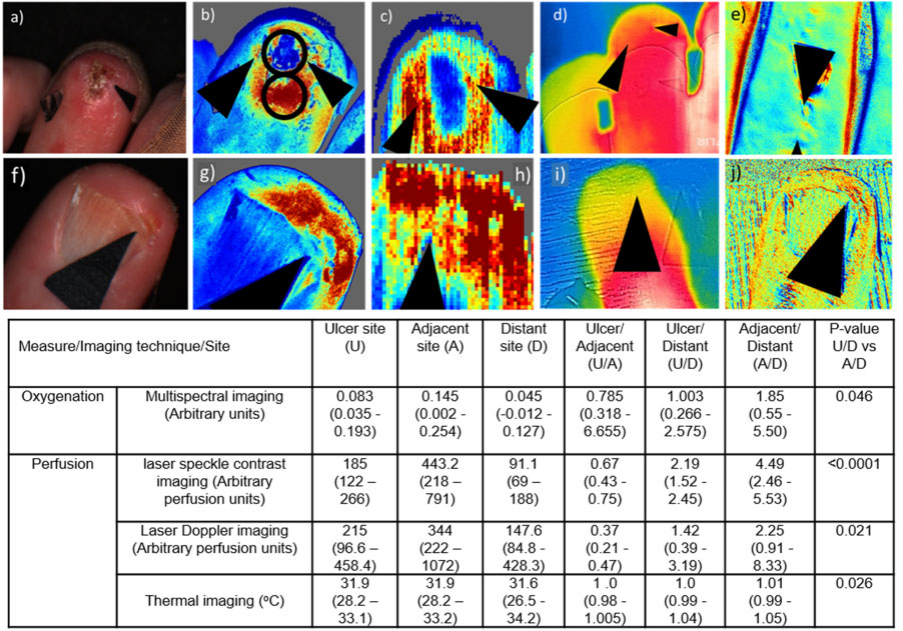
(**a**–**e**) extensor ulcer and (**f**–**j**) finger-tip ulcer; (**a**) and (**f**) photograph; (**b**) and (**g**) laser speckle contrast imaging; (**c**) and (**h**) laser Doppler image; (**d**) and (**i**) thermal image and (**e**) and (**j**) multispectral imaging. Arrows indicate ulcer site. For (**b**–**e**) and (**g**–**i**), blue represents relatively low perfusion/oxygenation and red high. (**b**) shows examples of the regions of interest from which data were taken at the ulcer and adjacent sites. Below: table of data from the imaging techniques at each site.

Measurements of oxygenation were extracted from images taken by a bespoke multispectral imager. Broadband (white) light was shone onto skin and several images were taken over multiple wavelengths. The amount of light absorbed by de-oxyhaemoglobin and oxyhaemoglobin within the skin differs across wavelengths. Thus, de-oxyhaemoglobin and oxyhaemoglobin each have their own unique absorption spectrum, allowing the oxygenation levels to be calculated (as described previously [8]).

Perfusion was measured using three imaging techniques: laser speckle contrast imaging (MOORFLPI-2, Moor Instruments, UK), which images the upper levels of the cutaneous microcirculation, laser Doppler imaging (MOORLDI2, Moor Instruments), which images more deeply (both upper and lower microvascular levels) and thermography (FLIR-one, Sweden), which measures skin temperature; providing a proxy for superficial (microvascular) and deeper (macrovascular) tissue perfusion. The lesion area size was measured from laser Doppler images on accompanying grey-scale images. A Spearman’s rho test was used to assess correlations. This study complied with the Declaration of Helsinki and was approved by the NW Research Ethics Committee 6. All participants gave written consent.

Eight fingertip and one extensor DU were imaged. A region of interest was defined at the site of the ulcer, in an adjacent area of skin and at a distant area, away from the ulcer. The distant site was assumed to be unaffected by microvascular changes associated with the lesion. The ratios of ulcer/distant (U/D) and adjacent/distant (A/D) express the differences at the ulcer and adjacent sites compared with an ‘unaffected’ site. Data are shown in Fig. 1. Oxygenation was imaged in only six of the nine ulcers due to technical reasons. Oxygenation was decreased for 33% (2/6) of ulcers compared with the distant site and increased in 100% (6/6) of adjacent *vs* distant sites; U/D *vs* A/D, *P* = 0.046. Perfusion as measured by laser speckle contrast imaging was decreased in the ulcer *vs* distant site for 11% (1/9) and all adjacent sites *vs* distant sites were increased (9/9); U/D *vs* A/D, *P* <0.0001. Perfusion measured by laser Doppler imaging was lower in 44% (4/9) of ulcer sites *vs* distant sites and perfusion was higher in 78% (7/9) of adjacent sites *vs* distant sites; U/D *vs* A/D, *P* =0.021. Thermal imaging identified 44% (4/9) of ulcer sites as lower in perfusion than the distant sites and 67% of adjacent sites had increased perfusion as compared with the distant site; U/D *vs* A/D, *P* =0.026.

The median lesion area was 3.4 [3.25–4.95] cm^2^. Oxygenation was associated with perfusion as assessed by laser Doppler imaging (*r* = 0.83, *P* =0.04) but not as assessed with laser speckle contrast imaging (*r* = 0.49, *P* =0.33) or with thermography (*r* = 0.20, *P* =0.70). Neither oxygenation (*r* = −0.14, *P* =0.79), nor perfusion [laser speckle contrast (*r* = 0.20, *P* =0.60), laser Doppler imaging (*r* = −0.14, *P* =0.72)] were associated with the size of the lesion.

In conclusion, oxygenation was reduced for some but not all ulcers, offering support to our first hypothesis, although we acknowledge that the number of ulcers studied was small. Oxygenation was increased in the adjacent areas of all ulcers indicative of possible inflammation. All perfusion imaging techniques were sensitive to differences in perfusion between the ulcer site and adjacent and distant areas when considering the distant site (U/D *vs* A/D), suggesting ulcers affect both superficial and deeper tissue perfusion. Oxygenation showed a strong relationship to perfusion as measured by laser Doppler imaging, suggesting that hypoxic ulcers are poorly perfused. As well as providing insights into pathogenesis, measuring ulcer oxygenation and perfusion may provide a biomarker of ulcer healing for novel therapies.

## References

[1] Hughes M , Herrick AL. Digital ulcers in systemic sclerosis. Rheumatology 2017;56:14–25. 27094599 10.1093/rheumatology/kew047

[2] Khimdas S , Harding S, Bonner A, Zummer B, Baron M, Pope J, Canadian Scleroderma Research Group. Associations with digital ulcers in a large cohort of systemic sclerosis: results from the Canadian Scleroderma Research Group registry. Arthritis Care Res 2011;63:142–9. 10.1002/acr.20336 20740608

[3] Bérezné A , Seror R, Morell-Dubois S et al Impact of systemic sclerosis on occupational and professional activity with attention to patients with digital ulcers. Arthritis Care Res 2011;63:277–85. 10.1002/acr.20342 20824802

[4] Murray AK , Moore TL, Wragg E et al Pilot study assessing pathophysiology and healing of digital ulcers in patients with systemic sclerosis using laser Doppler imaging and thermography. Clin Exp Rheumatol 2016;34(Supp. 100):100–5. 27749241

[5] Ruaro B , Sulli A, Smith V et al Short-term follow-up ofdigital ulcers by laser speckle contrast analysis in systemicsclerosis patients. Microvasc Res 2015;101:82–5. 26142117 10.1016/j.mvr.2015.06.009

[6] Mirasoglu B , Bagli BS, Aktas S. Hyperbaric oxygen therapy for chronic ulcers in systemic sclerosis – case series. Int J Dermatol 2017;56:636–40. 28233289 10.1111/ijd.13570

[7] Markus YM , Bell MJ, Evans AW. Ischemic scleroderma wounds successfully treated with hyperbaric oxygen therapy. J Rheumatol 2006;33:1694–6. 16881126

[8] Poxon I , Wilkinson J, Herrick A, Dickinson M, Murray A. Pilot study to visualise and measure skin tissue oxygenation, erythema, total haemoglobin and melanin content using index maps in healthy controls. In: Optical Diagnostics and Sensing XIV: Toward Point-of-Care Diagnostics, SPIE Proceedings, SPIE BiOS, San Francisco, California, United States, 2014 Volume 8951. 10.1117/12.2038571.

