# Corrigendum to: Systemic sclerosis-related digital ulcers; a pilot study of cutaneous oxygenation and perfusion

**DOI:** 10.1093/rheumatology/keaa681

**Published:** 1999-12-30

**Authors:** Elizabeth Marjanovic, Tonia L Moore, Joanne B Manning, Graham Dinsdale, Sarah Wilkinson, Mark R Dickinson, Ariane L Herrick, Andrea K Murray

**Affiliations:** 1 Division of Musculoskeletal and Dermatological, Salford Royal NHS Foundation Trust, Manchester Academic Health Science Centre; 2 Photon Science Institute; 3 Department of Physics and Astronomy, The University of Manchester, Manchester, UK

##  

In the originally published version of this manuscript, there was an error in the title. The title referred to: “Systemic sclerosis-related digital calcinosis; a pilot study of cutaneous oxygenation and perfusion”. This has now been corrected to online to: “Systemic sclerosis-related digital ulcers; a pilot study of cutaneous oxygenation and perfusion”.

